# Effect of the Angiotensin-Converting Enzyme (ACE) (I/D) Polymorphism in COVID-19 Patients and Their Healthy Contacts

**DOI:** 10.7759/cureus.38610

**Published:** 2023-05-05

**Authors:** Prishni Gupta, Eli Mohapatra, Suprava Patel, Lisie L Patnayak, Rachita Nanda, Seema Shah, Jessy Abraham, Ajoy Behera, Atul Jindal

**Affiliations:** 1 Biochemistry, All India Institute of Medical Sciences Raipur, Raipur, IND; 2 Pulmonary Medicine, All India Institute of Medical Sciences Raipur, Raipur, IND; 3 Paediatrics, All India Institute of Medical Sciences Raipur, Raipur, IND

**Keywords:** allele, angiotensin, polymorphism, ace, covid-19

## Abstract

Introduction

The quest to understand the pathophysiology behind the deleterious effects of the coronavirus disease 2019 (COVID-19) outbreak took a turn when involvement of the angiotensin converting enzyme (ACE) receptors in different organs, especially the lungs, could explain all the clinical manifestations and adverse events in patients. The I/D polymorphism in the ACE gene, having been attributed in various studies, was also seen to have an effect in this pandemic. Present study aimed to analyze the effect of this I/D mutation in COVID-19 patients and in their healthy contacts.

Methods

Patients with past history of COVID-19 infection and their healthy contacts were enrolled in the study after obtaining ethical clearance and informed consent. The polymorphism was studied by real-time polymerase chain reaction (PCR). Data was analyzed in SPSS version 20 (IBM Corp., Armonk, NY, USA). p value less than 0.05 was taken as significant.

Results

The allelic distribution followed the Hardy-Weinberg equilibrium, with the wild ‘D’ allele being dominant in the population. Between the case and controls, the mutant ‘I’ allele was observed more in the controls, and the association was statistically significant.

Conclusion

From the results of the present study, it could be concluded that while the wild ‘D’ allele led to higher chances of being affected with COVID-19, the polymorphism to ‘I’ allele was relatively protective in nature.

## Introduction

The pathophysiological process facilitating the most talked about virus in the history of viral diseases, coronavirus disease 2019 (COVID-19), drove the researchers to uncover the various already established pathways in a completely different light. The novel coronavirus was responsible for millions of deaths across the globe, leaving behind a trail of respiratory disabilities in another million, the respiratory system being the center of the massacre [1]. Amongst the pathways that stole the limelight, the entry of the virus through the interaction of its spike protein with the angiotensin converting enzyme 2 (ACE2) receptor managed to explain the clinical scenario in most patients [2,3]. The common pathway leading to the systemic failure rampant in COVID-19 was hypothesized to be due to the systemic expression of ACE2, which is present in most organs of the body [2]. As discussed in various studies, ACE1 controls ACE2 expression and activity by holding the levels of angiotensin II [4]. ACE2 forms angiotensin 1-7 (renin-angiotensin-aldosterone system [RAAS]) from angiotensin II [5]. While angiotensin II induces proinflammatory effects, angiotensin 1-7 has protective functions in the body [6]. The imbalance in the ACE1/ACE2 arms, owing to common genetic polymorphisms, could cause adverse outcomes in severe acute respiratory syndrome coronavirus 2 (SARS-CoV-2) pathogenesis [7].

The ACE gene, located on chromosome 17 q 35, consists of 26 exons [8]. The insertion/deletion genotypes of a 287-bp Alu element repeat sequence in intron 16 cause alternating splicing in ACE protein, with one active site for the ACE I allele and two active sites for the D allele [9]. This I/D polymorphism affects the levels of ACE, the deletion being associated with more severe forms and adverse outcomes [10] in various conditions, including COVID-19. Alternatively, it could be assumed that the II genotype acts as a protective characteristic, keeping patients safe from the complications of acute respiratory distress syndrome (ARDS). Thus, the present study aimed to determine the prevalence of the I/D polymorphism in COVID-19 patients and their contacts without the infection.

## Materials and methods

Study design and study subjects

In a cross-sectional case control study, conducted by the Department of Biochemistry, All India Institute of Medical Sciences (AIIMS) Raipur, from January 2022 to January 2023, post COVID-19 patients and their respective healthy contacts were recruited, complying to the ethical standards and good clinical practice guidelines. Ethical clearance was obtained from the institute's ethical committee. The records of patients positive for COVID-19 in the years 2020 and 2021 were collected from the Central Medical Records Department of AIIMS Raipur.

A total of 64 volunteers (32 cases and 32 controls) above the age of 18 years were contacted personally. Volunteers who were reverse transcription polymerase chain reaction (RT-PCR) positive during the first or second phase of the pandemic (cases) with family members living with them during the same time period but being RT-PCR negative (controls) were included in the study. People with ongoing malignancy, pregnancy and on medication with ACE inhibitors were excluded. 

The sample size was initially taken as convenient sampling with 40 cases and 40 controls as no specific prevalence data was found in this region. However, only 32 cases and 32 controls could be recruited due to non-responsiveness.

Data collection and sample collection

The essential demographic and clinical details of each participant were recorded in self-designed case record forms. Whole blood ethylenediamine tetraacetic acid (EDTA) samples (2ml) were collected from each participant after obtaining written informed consent and explaining the study in brief. Samples were stored at -20 degrees centigrade till further processing.

Sample processing and genotype analysis

Leukocyte human genomic DNA was extracted from the samples by a PureLink Genomic DNA Mini Kit (Invitrogen, Waltham, MA, USA). The single nucleotide polymorphism (SNP) was identified and allelic frequency determined by genotyping master mix and SNP genotyping assay kit by Taqman (Invitrogen). PCR was done in the BioRad CFX-96 Real-Time System (Hercules, CA, USA). The primer sequences used were -CCCATTTCTCTAGACCTGCTGCCT- for VIC and -ATACAGTCACTTTTATGTGGTTTC- for FAM. The VIC probe detected allele 2, the wild insertion type, while the FAM probe reported allele 1, the mutant Alu insertion type of the ACE I/D variant. Each assay was run separately on samples. A heterozygous sample amplified with both assays whereas homozygous samples amplified with only one assay. The results were visualized and analyzed digitally and then recorded for analysis.

Statistical analysis

Patients’ details were recorded and tabulated in Excel 2016 (Microsoft, Redmond, WA, USA) and results were analyzed by SPSS version 20 (IBM Corp., Armonk, NY, USA). Continuous variables were checked for normality. Normally distributed parameters were represented as Mean ± SD and compared by student t test. Non-parametric data were reported as Median (Interquartile Range [IQR]) and analyzed by Mann-Whitney U test. Categorical data were presented as percentages and compared by Chi-Square test. The allele and genotype frequencies were compared by Fisher's exact. Odds ratio and logistic regression model were performed to measure the strength of associations. A p value of less than 0.05 was taken significantly.

## Results

The clinic-demographic variable and allelic frequency were compared between the 32 cases and 32 controls recruited in the study.

The clinical and demographic distributions

The gender, age, present and past clinical histories, and the practices undertaken by the study participants during the COVID-19 infection were compared between the cases and controls (Table 1).

**Table 1:** The clinico-demographic variables compared between the cases and controls

Parameters	Study participants		Chi Square test		Univariate analysis	
	Cases (n=32)	Controls (n=32)	χ2	p value	Odds ratio	p value
Demographics and practices of volunteers						
Gender						
Male	23	11	9.035	0.005	4.878	0.003*
Female	9	21				
Age						
< 45 years	22	21	0.070	0.791	1.152	0.266
≥ 45 years	10	11				
Isolation of patients						
Yes	16	8	4.267	0.070	0.333	0.041
No	16	24				
Use of mask						
Yes	16	11	1.602	0.311	0.524	0.208
No	16	21				
Clinical history of volunteers						
Diabetes Mellitus						
Yes	2	3	0.217	1.000	1.552	0.645
No	30	29				
Hypertension						
Yes	3	3	0.000	1.000	1.000	1.000
No	29	29				
Lung disorders						
Yes	0	0	-	-	-	-
No	32	32				
Obesity						
Yes	7	8	0.087	1.000	1.190	0.295
No	25	24				
Chronic Kidney Disease						
Yes	0	0	-	-	-	-
No	32	32				
Heart conditions						
Yes	0	0	-	-	-	-
No	32	32				
Chronic Liver Disease						
Yes	0	0	-	-	-	-
No	32	32				
Cancer						
Yes	0	0	-	-	-	-
No	32	32				

The ACE gene at the rs1799752 has two reported allelic distribution, the mutant polymorphisms of insertion of Alu element denoted by ‘I’ or the wild deletion allele represented as ‘D’. The two genotypes reported in the present study were homozygous II and homozygous DD. The distribution of the genotypes in the two participant groups with the analysis is shown in Table 2.

**Table 2:** Analysis between the genotypes and volunteer groups

Participants	Genotype		
	I/I	D/D	Total
Case	12	20	32
Control	19	13	32
Total	31	33	64
Chi-square test			
χ2	3.065		
p value	0.133		
Univariate analysis			
Odds ratio	2.435		
p value	0.082		
Multivariate analysis			
Odds ratio	1.222		
p value	0.043*		

Hardy-Weinberg equilibrium

p = frequency of I = 48.44%

q = frequency of D = 51.56%

p2+ q2 + 2pq = (64/128)2 + (66/128)2 + 2(64/128)(66/128) = 0.23 + 0.27 + 0.50 = 1

The result of the Hardy-Weinberg equation indicates that the genotype distribution complies with the Hardy-Weinberg equilibrium for the genotyping distribution in a population. The genotype and allele distribution also shows that the allele ‘D’ is the dominant allele in the study population.

Pearson correlation

On applying a Pearson correlation analysis test between the genotypes and participant groups, it was seen that there was a negative correlation, however the p value was not significant (p = 0.08).

Clinical and demographic distributions in the two genotype combinations

Table 3 gives the results of the comparisons between the two genotypes and gender practices adopted during COVID-19 and the participants’ past and present histories.

**Table 3:** The clinico-demographic variables compared between the genotypes II and DD

Parameters	Genotype		Chi Square test		Univariate analysis	
	I/I	D/D	χ2	p value	Odds ratio	p value
Demographics and practices of volunteers						
Gender						
Male	14	16	0.71	0.808	0.937	0.267
Female	17	17				
Age						
< 45 years	22	21	0.008	0.928	0.952	0.091
≥ 45 years	11	10				
Isolation of patients						
Yes	12	12	0.038	1.000	1.105	0.194
No	19	21				
Use of mask						
Yes	12	15	0.298	0.621	0.757	0.584
No	19	18				
Clinical history of volunteers						
Diabetes Mellitus						
Yes	2	3	0.155	0.694	1.450	0.391
No	29	30				
Hypertension						
Yes	2	4	0.605	0.673	2.000	0.766
No	29	29				
Lung disorders						
Yes	0	0	-	-	-	-
No	31	33				
Obesity						
Yes	8	7	0.188	0.771	0.774	0.433
No	23	26				
Chronic Kidney Disease						
Yes	0	0	-	-	-	-
No	31	33				
Heart conditions						
Yes	0	0	-	-	-	-
No	31	33				
Chronic Liver Disease						
Yes	0	0	-	-	-	-
No	31	33				
Cancer						
Yes	0	0	-	-	-	-
No	31	33				

## Discussion

Present study compared the ACE II/DD genotype polymorphism between COVID-19 patients and their healthy contacts who lived with them during the epidemic time. As observed in Table 1, males were infected more than the females in the study population, with a significant association and odds ratio. Patient practices like isolation of patients and use of masks did not show significant association with cases and controls.

Patients’ conditions like diabetes mellitus, hypertension, lung disorders, obesity, chronic kidney disease, cardiac conditions, chronic liver diseases and cancer also did not show any significant association between cases and controls.

The genotype frequency was compared between the cases and controls by chi-square, univariate and multivariate analysis (Table 2). Though the association and univariate odds ratio were not statistically significant, the number of cases with the wild DD genotype were higher, while the controls showed more numbers of the II mutant genotype. The correlation analysis result showed a negative correlation between the II genotype and COVID-19 infection. The result of the Hardy-Weinberg equation indicated that the I/D polymorphism followed the rules of the Hardy-Weinberg equilibrium of genotypes. The multivariate logistic regression analysis gave a significant odds ratio, statistically proving that the wild DD genotype was more prone to COVID-19 infection, while the mutant II genotype was relatively protective. Comparing the clinic demographic parameters amongst the genotypes did not show any significant association. The genotypes were almost equally distributed between the female and male genders.

The ACE I/D polymorphism has been extensively studied, correlating with various studies. The ACE I/D polymorphism has been associated with conditions like vitiligo [11], different cancers [12], infertility and loss of pregnancies [13], cerebrovascular accidents, diabetic nephropathy and other renal disease [14,15], and cardiovascular complications [16]. Along with them, the DD genotype has also been associated with increased mortality in ARDS patients in several studies [17].

The fact that the wild ‘D’ allele affects the respiratory patients adversely led to its inclusion in the hypothesis of COVID-19 severity, as the underlying pathogenesis via the ACE2 receptor got accepted widely. Several studies reported the association of the DD genotype with high mortality and increased severity in COVID-19 patients. Cecilia et al. in their study on I/D polymorphism and acute pulmonary embolism in COVID-19 pneumonia patients concluded that the DD homozygous polymorphism was significantly higher in the patients with pulmonary embolism than in patients without the complication [18]. Livshits et al. studied the prevalence of ACE I/D polymorphism in a few European countries along with Ukraine [19]. They found that there was a negative correlation between the II genotype and chances of SARS-CoV-2 infection. In another study, conducted by Verma et al., the impact of the I/D polymorphism on the severity of COVID-19 patients was evaluated. They concluded that this polymorphism could be used as a predictor for severity in COVID-19 patients in India [20]. The results of a meta-analysis done by Hatami et al. on the worldwide ACE I/D polymorphism effect on COVID-19 recovery demonstrated that an increase in I/D allele frequency ratio increased the recovery rate significantly [21]. Another meta-analysis covering the studies on ACE gene I/D polymorphism and severity of SARS-CoV-2 infection in hospitalized patients by Oscanoa et al. reported that patients with the dominant homozygous DD genotype were at 47% higher risk of severe COVID-19 as compared to the mutant II genotype [22].

The results of the present study were complimentary to discussed studies done on COVID-19 though the present study had a few limitations, like small sample size and retrospective study design that led to recall as a confounding factor in data collection. However, the present study was one of its kind in this region and could be the foundation for future studies to understand the protective role of the I/I genotype of the ACE gene.

## Conclusions

Present study was a first of its kind in central India, emphasizing the role of the I/D polymorphism in COVID-19 patients and their contacts who did not get infected. Though the limitations of the study handicapped the results mildly, the present study demonstrated that while the dominant wild deletion ‘D’ allele of the ACE gene could cause a higher susceptibility to COVID-19 infection, the mutant insertion ‘I’ allele could be protective and decrease the chances of infection even in individuals coming in direct contact with patients. Further studies with larger sample size are needed, to finalize the role of the I/D polymorphism in not only COVID-19 but also other disorders involving the ACE gene.
